# Supplementary material for: Multiple poliovirus-induced organelles suggested by comparison of spatiotemporal dynamics of membranous structures and phosphoinositides
Source: PLoS Pathog. 2018 Apr 27;14(4):e1007036. doi: 10.1371/journal.ppat.1007036 (PMC5942851; doi:10.1371/journal.ppat.1007036)
Supplement: S1 Fig — (A,B) Kinetics of RNA replication of subgenomic replicon RNA by WT (panel A) and EG (panel B) as measured by luciferase activity and Northern blotting. Values are represented as percent of the maximum value from each respective assay. (PDF) [file ppat.1007036.s001.pdf]

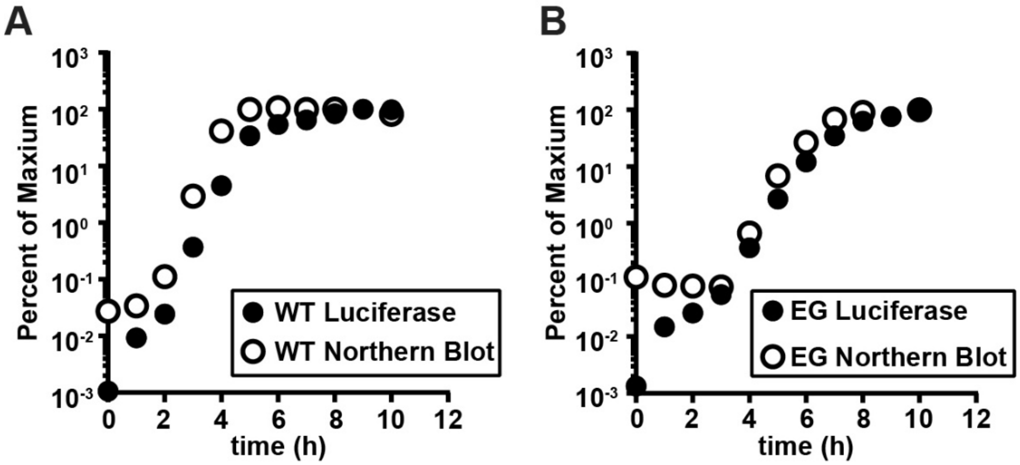

S1 Fig. **Comparison of the kinetics of luciferase activity and RNA accumulation by Northern blotting for WT and EG subgenomic replicon.** (**A,B**) Kinetics of RNA replication of subgenomic replicon RNA by WT (panel A) and EG (panel B) as measured by luciferase activity and Northern blotting. Values are represented as percent of the maximum value from each respective assay.
