# Supplementary material for: Multiple poliovirus-induced organelles suggested by comparison of spatiotemporal dynamics of membranous structures and phosphoinositides
Source: PLoS Pathog. 2018 Apr 27;14(4):e1007036. doi: 10.1371/journal.ppat.1007036 (PMC5942851; doi:10.1371/journal.ppat.1007036)
Supplement: S2 Fig — Kinetics of luciferase activity as measured for WT, EG and GG subgenomic replicon RNAs in the presence of the replication inhibitor, GuHCl. A subgenomic replicon in which the polymerase was inactivated (Pol minus) was also included as a control. HeLa cells were transfected with in vitro transcribed replicon RNA, placed at 37°C in the absence (Pol minus) or presence of 3 mM GuHCl (WT, EG and GG) and luciferase activity (RLU/μg) measured at the indicated times post-transfection. Data are represented as means ± SEM. n = 3. (PDF) [file ppat.1007036.s002.pdf]

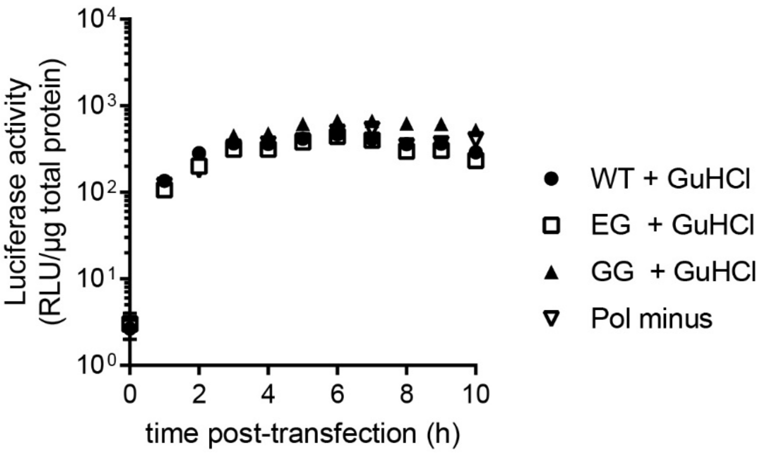

S2 Fig. **The initial translation of WT, EG and GG subgenomic RNA replicons are the same in the presence of a replication inhibitor.** Kinetics of luciferase activity as measured for WT, EG and GG subgenomic replicon RNAs in the presence of the replication inhibitor, GuHCl. A subgenomic replicon in which the polymerase was inactivated (Pol minus) was also included as a control. HeLa cells were transfected with *in vitro* transcribed replicon RNA, placed at 37 °C in the absence (Pol minus) or presence of 3 mM GuHCl (WT, EG and GG) and luciferase activity (RLU/µg) measured at the indicated times post-transfection. Data are represented as means ± SEM. n= 3.
