# Supplementary material for: Multiple poliovirus-induced organelles suggested by comparison of spatiotemporal dynamics of membranous structures and phosphoinositides
Source: PLoS Pathog. 2018 Apr 27;14(4):e1007036. doi: 10.1371/journal.ppat.1007036 (PMC5942851; doi:10.1371/journal.ppat.1007036)
Supplement: S3 Fig — Virus yield (PFU per mL) plotted as a function of time post-infection from cells infected at different MOIs (10, 25, 50 and 100). The initial differences in virus yield at early times is a direct reflection on the input MOI. At 6 and 8 h post-infection no substantial differences in yield of virus are observed. Data are represented as means ± SEM. n = 3. (PDF) [file ppat.1007036.s003.pdf]

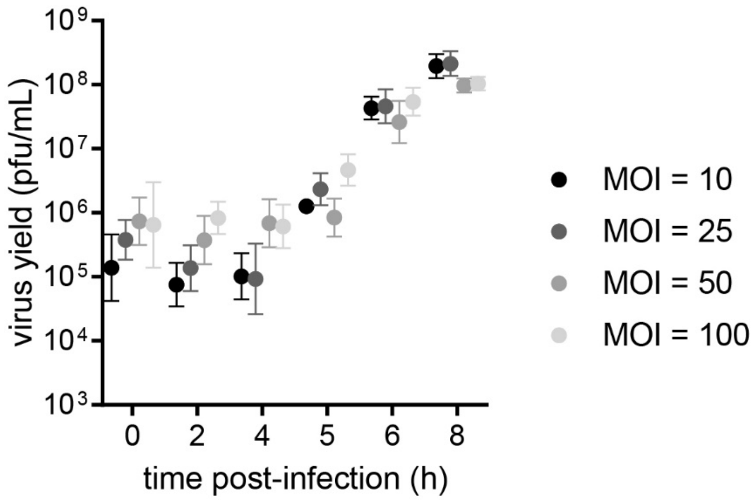

S3 Fig. **Higher MOI does not substantially increase virus production**. Virus yield (pfu per mL) plotted as a function of time post-infection from cells infected at different MOIs (10, 25, 50 and 100). The initial differences in virus yield at early times is a direct reflection on the input MOI. At 6 and 8 h post-infection no substantial differences in yield of virus are observed. Data are represented as means ± SEM. n= 3.
